# Trauma‐informed patient and public‐engaged research: Development and evaluation of an online training programme

**DOI:** 10.1111/hex.13668

**Published:** 2022-11-16

**Authors:** Amber M. Gum, Mary Goldsworthy, Lucy Guerra, Alison Salloum, Meredith Grau, Sheri Gottstein, Carol Horvath, Annanora Fields, Johnny Crowder, Robb Holley, Leigh J. Ruth, Karim Hanna

**Affiliations:** ^1^ myPATH Partnership University of South Florida Tampa Florida USA; ^2^ Department of Mental Health Law & Policy University of South Florida Tampa Florida USA; ^3^ Department of Internal Medicine University of South Florida Tampa Florida USA; ^4^ School of Social Work University of South Florida Tampa Florida USA; ^5^ Crisis Center of Tampa Bay Tampa Florida USA; ^6^ Department of Psychiatry and Behavioral Neurosciences University of South Florida Tampa Florida USA; ^7^ Department of Family Medicine University of South Florida Tampa Florida USA

**Keywords:** co‐production, participatory research, patient and public involvement and engagement, stakeholder‐driven research, training, trauma, trauma‐informed care

## Abstract

**Introduction:**

As patients, members of the public, and professional stakeholders engage in co‐producing health‐related research, an important issue to consider is trauma. Trauma is very common and associated with a wide range of physical and behavioural health conditions. Thus, it may benefit research partnerships to consider its impact on their stakeholders as well as its relevance to the health condition under study. The aims of this article are to describe the development and evaluation of a training programme that applied principles of trauma‐informed care (TIC) to patient‐ and public‐engaged research.

**Methods:**

A research partnership focused on addressing trauma in primary care patients (‘*my*PATH’) explicitly incorporated TIC into its formation, governance document and collaborative processes, and developed and evaluated a free 3‐credit continuing education online training. The training was presented by 11 partners (5 professionals, 6 patients) and included academic content and lived experiences.

**Results:**

Training participants (*N* = 46) positively rated achievement of learning objectives and speakers' performance (ranging from 4.39 to 4.74 on a 5‐point scale). The most salient themes from open‐ended comments were that training was informative (*n* = 12) and that lived experiences shared by patient partners were impactful (*n* = 10). Suggestions were primarily technical or logistical.

**Conclusion:**

This preliminary evaluation indicates that it is possible to incorporate TIC principles into a research partnership's collaborative processes and training about these topics is well‐received. Learning about trauma and TIC may benefit research partnerships that involve patients and public stakeholders studying a wide range of health conditions, potentially improving how stakeholders engage in co‐producing research as well as producing research that addresses how trauma relates to their health condition under study.

**Patient or Public Contribution:**

The *my*PATH Partnership includes 22 individuals with professional and lived experiences related to trauma (https://www.usf.edu/cbcs/mhlp/centers/mypath/); nine partners were engaged due to personal experiences with trauma; other partners are community‐based providers and researchers. All partners contributed ideas that led to trauma‐informed research strategies and training. Eleven partners (5 professionals, 6 patients) presented the training, and 12 partners (8 professionals, 4 patients) contributed to this article and chose to be named as authors.

## 1 INTRODUCTION

Patient and public involvement and engagement (PPIE) in co‐producing health‐related research are growing rapidly on an international scale, with efforts across numerous countries recently described in a special issue of *The BMJ* ^1^ and other reviews.^2^, ^3^, ^4^, ^5^ In the United States, the Patient‐Centered Outcomes Research Institute (PCORI)^6^ was created in 2012 to build the nation's capacity, production and dissemination of stakeholder‐driven research. (‘Stakeholder‐driven research’ is a phrase commonly used by PCORI and is conceptually similar to phrases such as PPIE as applied to research, co‐production of research, community‐based participatory research or participatory action research.) As groups of patients, members of the public, and professional stakeholders join to co‐produce research, they would likely benefit from attending to an important issue that is relevant across a wide range of health conditions—trauma. Traumatic events are very common, impacting 54%–74% of adults globally,^7^, ^8^ which has implications for how PPIE‐based research partnerships function as well as the health condition they are studying. First, among patients with health conditions and members of the public who become engaged in co‐producing research, many of them will have experienced one or more traumatic events, given how common these events are for individuals living with chronic health conditions and in the public. This lived experience of trauma can make it difficult for some individuals to feel safe and empowered to fully engage as research partners. Second, trauma complicates the identification, course and treatment for many health conditions,^9^, ^10^, ^11^ so research groups may want to incorporate trauma into their research agendas.

To address these challenges, our stakeholder‐driven research partnership aimed to develop and evaluate training about the application of trauma‐informed principles to stakeholder‐driven research, as part of a PCORI‐funded capacity‐building project.

### 1.1 The relevance of trauma to stakeholder‐driven research partnerships

Trauma is highly prevalent in the general population^7^, ^8^ and places individuals at risk of a wide range of health conditions (e.g., cardiovascular disease, diabetes, cancer, respiratory disease, substance misuse, depression, anxiety),^7^, ^12^, ^13^, ^14^, ^15^, ^16^, ^17^, ^18^, ^19^, ^20^, ^21^, ^22^ which suggests that research partnerships will engage many people impacted by trauma, whether intending to or not. Trauma is defined as ‘an event, series of events, or set of circumstances that is experienced by an individual as physically or emotionally harmful or life‐threatening and that has lasting adverse effects on the individual's functioning and mental, physical, social, emotional, or spiritual well‐being’ (p. 7).^23^ Trauma includes a range of experiences, such as child abuse (i.e., physical, emotional or sexual) and neglect, potentially other adverse childhood experiences (e.g., unstable home environment),^13^ domestic violence, physical or sexual assault, combat or war, serious accidents and injuries, natural disasters, witnessing trauma or cultural trauma.^23^

Globally, most adults have experienced at least one traumatic event, with estimates across countries ranging from 54% (Spain) to 74% (South Africa),^7^ for a global estimate of 70.4% across 24 countries.^7^ Over half (60%) of American adults have experienced at least one traumatic event.^8^ Among people with at least one traumatic exposure, the mean number of traumatic events is 4.6 (chronic traumas such as repeated child abuse were counted as one event),^24^ and individuals exposed to childhood trauma (e.g., abuse, neglect) are at increased risk of further trauma exposure as adults.^24^, ^25^

Trauma exposure is a potent risk factor for many deleterious effects. Globally, of those exposed to traumatic events, 5.6% develop posttraumatic stress disorder (PTSD), which is persistent for approximately half.^26^ Beyond PTSD, trauma exposure increases the risk of many other outcomes—poor health behaviours (e.g., smoking, not eating well or exercising, risky sexual behaviours); numerous physical health problems and physical disability (e.g., cardiovascular disease, diabetes, respiratory disease, cancer, autoimmune conditions); other behavioural health conditions (e.g., anxiety, depression, substance misuse); relationship problems and death, including suicide and all‐cause mortality.^7^, ^12^, ^13^, ^14^, ^15^, ^16^, ^17^, ^18^, ^19^, ^20^, ^21^, ^22^ In the general population, death by suicide has been found to be over five times higher for individuals with PTSD compared to those without PTSD.^27^ Individuals with trauma histories use more healthcare services,^28^ and trauma is estimated to cost $748 billion annually in health‐related outcomes in North America.^19^ Patients with posttraumatic stress symptoms tend to show poorer adherence to medical regimens and worse physical health outcomes.^9^, ^10^, ^11^ This situation has worsened since the onset of the COVID‐19 pandemic, with traumatic events increasing for the public^29^, ^30^, ^31^, ^32^ and healthcare workers.^33^ Addressing and treating posttraumatic effects has mental, as well as physical, benefits; a systematic review has shown that treating PTSD improves not only mental health, but also physical health, including cardiovascular, diabetic and metabolic outcomes.^34^

Therefore, it will likely benefit many research partnerships to learn more about trauma and consider its impact on their partners and relevance to the health condition under study, although some preparation of the partnership is warranted when beginning this process. Some trauma survivors often feel unsafe, disempowered and dysregulated (i.e., hyperarousal or hypoarousal).^36^ These characteristics could make it difficult for some partners to discuss or learn about trauma; these characteristics also could exacerbate power differentials that already occur between patient and family stakeholders with professional stakeholders. Trauma‐informed care (TIC) is an organizational model designed to address these types of challenges across the diverse community, educational, health and social service settings that could be applied to research partnerships.

### 1.2 Overview of TIC

TIC aims to promote a sense of safety, collaboration and empowerment for trauma survivors and all stakeholders in an organization to promote healing. It ‘*realizes* the widespread impact of trauma and understands potential paths for recovery; *recognizes* the signs and symptoms of trauma in clients, families, staff, and others involved with the system; and *responds* by fully integrating knowledge about trauma into policies, procedures, and practices, and seeks to actively *resist* re‐traumatization’ (p. 9).^23^ TIC involves six principles: safety, trustworthiness and transparency, peer support, collaboration, empowerment and humility and responsiveness.^23^ A recent systematic review of 23 studies across settings (e.g., schools, behavioural health service settings) found that trauma‐informed staff training improved staff knowledge, attitudes and behaviours in some studies, and five studies found positive impacts on student or patient outcomes.^36^

Application and research on TIC in healthcare settings is in the early stage. A general model of trauma‐informed healthcare involves a foundation of TIC knowledge among all personnel, a calm and empowering environment, educating patients and personnel about trauma and its relationships with health, inquiring about patients' trauma histories and responding appropriately to patients' disclosures of trauma.^12^ In addition to its hypothesized benefits for patients, TIC promotes awareness of trauma among personnel and encourages self‐care; as such, TIC is thought to benefit staff well‐being,^24^ although empirical evidence is lacking. TIC principles and practices have also been applied to community outreach, policy,^38^, ^39^ research participants^40^, ^41^ and a research advisory board,^42^ but no publications were identified that developed and evaluated training applying TIC principles to PPIE‐based research partnerships.

### 1.3 myPATH partnership and aims

Because our research partnership explicitly addresses the topic of trauma, we applied the principles and practices of TIC to its initial development and continuation over the past 4 years. Our partnership is called *my*PATH—a *patient‐centered* Partnership Addressing Trauma and Healing. *my*PATH's mission is to ‘sustain a partnership of patients, providers, researchers, and other stakeholders, that will collaboratively develop and conduct research to: (1) integrate principles of trauma‐informed care into primary care settings; and (2) deliver personalized interventions to patients with trauma histories that will impact outcomes meaningful to patients’.^42^ *my*PATH partners have met monthly since November 2017, during which time we engaged in co‐learning about trauma, developed a governance document, developed and implemented trauma‐informed research practices, developed live and online training applying TIC to research partnerships, conducted research related to the COVID‐19 pandemic, conducted surveyed 249 stakeholders to plan research and submitted a grant proposal involving interventions to address trauma in primary care settings. These efforts have been supported by two PCORI contracts. For this article, we aimed to describe the development and evaluation of the training.

## 2 METHODS

### 2.1 Development of training content, format and processes

#### 2.1.1 Partnership development and capacity‐building

The training was based on the partnership's experience forming and sustaining the partnership over the first 2 years. The partnership was formed through networking, beginning with an academic psychologist with expertise in behavioural health integration in primary care and an academic internal medicine physician, adding other professionals with expertise in trauma and healthcare and selecting and inviting individuals who had engaged in healthcare or social services related to traumatic experiences. Care was taken to engage individuals from various professional disciplines and patient representatives with diverse traumatic experiences. The application of TIC principles began in the initial discussions inviting patient representatives to join the partnership, by (a) discussing its purpose; (b) conveying the importance of learning from each person's perspective; (c) assuring they would not be required to disclose personal experiences, they could do whatever was needed for self‐care if any discussions became difficult, and they could resign at any time and (d) encouraging them to take as much time as needed and ask questions to facilitate an informed, uncoerced decision regarding whether to join the partnership.

The initial partnership included 10 founding members. The five professionals' disciplines included psychology, internal medicine, nursing and mental health counselling. The five patient partners had experienced various types of traumas, including child abuse, workplace trauma and physical and sexual assault. It was soon recognized that patient partners also contributed professional expertise, including childcare, healthcare, social services and entrepreneurship. Nine partners were female, and one was male; partners represented a range of ages (from early 20s to 70s) and racial and ethnic groups (e.g., white, Latina/Latino, Black/African‐American, Asian, American Indian). (Partners have not been formally surveyed regarding their demographic characteristics or trauma experiences, given the partnership's decision, as documented in the governance document, to not require partners to disclose personal or traumatic experiences. Thus, the descriptions of partners' characteristics in this manuscript are based on public information and partners' voluntary statements during meetings.)

These initial partners secured funding from a PCORI Pipeline to Proposal award (October 2017–2018), which provided compensation for all professional and patient partners to meet monthly and develop infrastructure. During the first year of meetings, partners focused on rapport‐building; co‐learning about trauma, TIC and stakeholder‐driven research and developing a governance document. Partners learned about trauma and TIC through brief presentations by partners; review and discussion of readings and other resources regarding trauma and TIC as presented by the US Substance Abuse and Mental Health Services Administration (SAMHSA), Trauma‐Informed Care Implementation Resource Center and experts in trauma in primary care^23^, ^43^, ^44^, ^45^ and general discussion among partners. Incorporating elements of TIC, initial meetings also included discussions about the nature of trauma, difficulties discussing trauma, limitations of what would or would not be discussed and strategies for managing difficult emotions that might arise during meetings or when reviewing materials. Partners also reviewed resources related to community‐based participatory research^46^, ^47^ and stakeholder‐driven research.^48^ These resources and discussions led to the governance document, which included elements suggested by PCORI, including vision and mission, membership requirements and expectations, communication, decision‐making and values. The governance document also included the results of our discussions about trauma. As summarized in the governance document (available from the first author), it was determined that ‘trauma’ has a subjective component, partners would not be required to disclose their personal experiences, no subjects were off‐limits if discussions remained respectful and considerate, and partners could engage in various self‐care behaviours if they became distressed during a discussion.

Partners also identified additional capacity‐building needs, which resulted in a second PCORI proposal being submitted and awarded (January 2020 to December 2021). Its first aim was to expand the partnership and network connections (22 partners currently), by intentionally considering professional disciplines, networks, trauma‐related and demographic characteristics that were not represented in the original 10‐member partnership. New professional partners were added from psychiatry, family medicine, social work, substance use treatment and healthcare administration; and they represented multiple networks across Florida, including the state unit of a mental health advocacy organization, a regional agency that contracts with over 100 behavioural health agencies and another state university's academic medical centre. New patient partners were added by connecting with other organizations (e.g., healthcare organizations serving veterans and LGBTQ+). New patient partners also added new areas of professional expertise, including television production, music, marketing, peer counselling and veterans services. The current partnership includes 16 females and 6 males with a similar range in age, race and ethnicity as the founding group.

#### 2.1.2 Training development and pilot‐testing

The second aim of the second PCORI contract was to develop, deliver and evaluate training on TIC and trauma‐informed, stakeholder‐driven research. The initial training content was developed collaboratively by the partnership through discussion across nine monthly meetings, by discussing potential topics and learning objectives, reviewing websites and resources, reviewing presentation platforms and discussing format and speakers. The training content was divided into three components: (a) an overview of trauma and TIC; (b) an overview of stakeholder‐driven research and (c) an application of TIC to stakeholder‐driven research. Patient partners noted that personal stories can be more compelling than academic, research‐based content alone. Thus, partners were invited to share personal experiences, but it was emphasized that no partner should feel obligated to present, or if they chose to present, that they should not feel obligated to present details they would prefer to omit. Eleven partners (five professionals and six patients) volunteered to present a portion of the training content. Those who presented personal statements elected to prerecord these statements; one patient partner who was a television news executive producer arranged for a professional videographer to video‐record these statements.

Regarding format, partners planned to offer the training in two phases: a two‐part live training workshop that would involve the partners and a small number of stakeholders, followed by enduring online training. Due to the COVID‐19 pandemic, partners changed the format of the live training workshops from in‐person to synchronous videoconference. Partners elected to use Prezi as the presentation software, given its features that promote engagement with online content. Partners also selected Moodle for housing the online training, given that one goal of the training was to build sustained relationships with more stakeholders, and Moodle requires a login and email address to access the training. These decisions were led by patient partners after academic partners presented options.

Partners established processes by which several types of professionals (i.e., physicians, advanced practice providers, nurses, social workers, psychologists, mental health counsellors and peer specialists) could earn free continuing education units (CEUs). This process was initiated after a patient partner inquired whether offering free CEUs would be possible; this patient partner had observed at a professional conference that sessions offering CEUs were particularly well‐attended. Research partners worked with two groups of university CEU providers (one for health professionals and one for social service and behavioural health professionals), reallocated the budget for CEUs and completed all required CEU forms (e.g., learning objectives), obtaining feedback and approvals from all partners at each monthly meeting (e.g., reviewing proposed budget reallocations before implementing changes).

Twenty‐one individuals (including partners who presented a portion of the training) participated in the live videoconference training in November 2020 (21 for Part 1, 17 for Part 2), and 13 completed anonymous online evaluations. At the monthly meeting following the live training, the results of these evaluations were reviewed, and revisions were planned. The feedback was largely positive, and suggestions primarily focused on improving the audiovisual quality of the training.^50^ After changes were made to the presentation materials, the academic presenters rerecorded their video presentations for the final online training.

### 2.2 Final online enduring training

#### 2.2.1 Training content

As stated previously, the training content was divided into three components, all available on a Moodle website. Part 1 involved watching a 60‐min video, in which partners provided an overview of trauma, the prevalence of trauma in the United States, the impacts of trauma on physical and behavioural health, the importance of resilience and the principles and practices of TIC. This video summarized: (a) research findings from the seminal ACE Study,^13^, ^14^ updated with recent research on physical and behavioural health impacts of trauma and resilience^15^, ^16^, ^17^, ^18^, ^19^, ^20^, ^21^, ^22^, ^29^; (b) SAMHSA's TIC model^24^ and (c) Machtinger et al.s'^12^, ^44^ model of TIC in healthcare. This information was presented by four different academic partners. This video also incorporated personal statements from patient partners about aspects of their trauma and healthcare experiences they wished to convey.

Part 2 utilized PCORI resources about stakeholder‐driven research, which learners read and interacted with at their own pace. PCORI has developed a Research Fundamentals Learning Package, with two introductory sections and five modules that help stakeholders new to research learn about the health research process and how to become fully involved as a stakeholder. Two sections were included as part of our training package: ‘Engaging in Stakeholder‐Driven Research’^51^ and ‘Developing Research Questions: Module 1’.^52^ The first of these sections is an interactive video that includes different types of stakeholders, including patient representatives, that provides an introduction to stakeholder‐driven research. The second section provides an overview of how to begin developing research questions and designing a research study. While not directly related to trauma, this segment of the training was included to provide all participants with encouragement, basic concepts and strategies for becoming engaged in research. One aim of our partnership was to build a larger collaboration including members of the public and community‐based professionals, who would then have opportunities to provide input regarding research being planned by the partnership.

**Figure 1:**
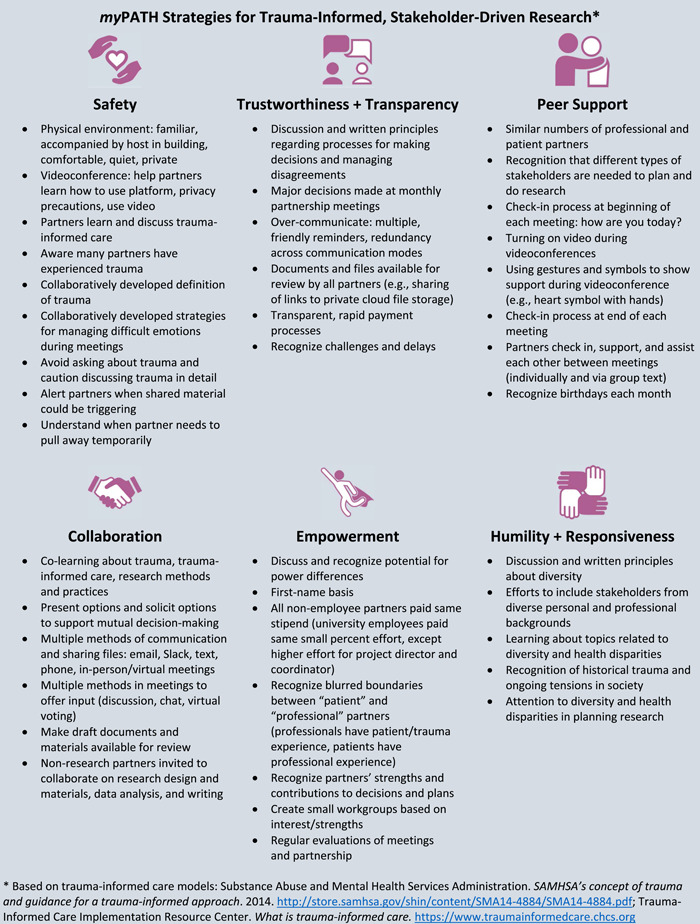
*my*PATH strategies for trauma‐informed, stakeholder‐driven research

Part 3 involved viewing a 60‐min video, in which *my*PATH partners described strategies for applying TIC principles to stakeholder‐driven research partnerships, organized by the six TIC principles. These strategies are summarized in Figure 1 and below. This section was presented by the first and second authors (the Project Director and Coordinator, respectively), incorporating video‐recorded statements by patient partners regarding their experiences as research collaborators.

As described in Part 3, to foster *safety*, strategies involve creating a comfortable physical environment, privacy precautions for videoconference meetings (e.g., private link to enter, verifying the identity of participants, using video as much as possible) and agreed‐upon guidelines for discussing the trauma and managing distress. Partners attempt to avoid trauma triggers by not asking other partners detailed personal questions, not discussing traumatic topics in graphic detail and presenting warnings when sharing resources that might be distressing. Partners are encouraged to engage in self‐care as needed during meetings, such as stepping away or ‘zoning out’ temporarily if a difficult topic arises.

Many of the strategies related to other TIC principles are consistent with general principles and strategies for stakeholder‐driven research,^53^ particularly strategies for *trustworthiness and transparency, collaboration, empowerment, humility, and cultural responsiveness* (Figure 1). Given the overlap between these TIC principles and stakeholder‐driven research principles, many research partnerships may already be implementing some strategies that foster TIC, without realizing it. Strategies that other research partnerships may already be using include developing communication and decision‐making guidelines, sharing information and documents, budget transparency, first‐name basis and other strategies to minimize power differentials and attending to diversity and health disparities.

*Peer support* is a value emphasized in TIC, and several strategies were included to build and sustain relationships among partners, including a brief check‐in at the beginning and end of each meeting, which is a common practice in trauma‐informed organizations. Another noteworthy strategy has been the recognition of the blurred boundaries between ‘patient’ and ‘professional’ partners—many ‘professional’ partners have had personal (and work‐related) traumatic experiences, and ‘patient’ partners also advance our work through their professional experiences and skills. We have found small workgroups and projects to be an effective way to actively engage patient partners, such as in the video‐recorded statements for the training and a branding workgroup that developed the *my*PATH name and logo.

Regarding *humility and responsiveness*, the strategies applied to our research partnership included a statement about diversity across a variety of domains in the governance document; intentional discussions about stakeholder perspectives that needed to be added to the partnership; learning about diversity, health disparities and cultural trauma and attending to diversity in planning research (Figure 1). The training included a brief overview of these strategies as well as research findings regarding trauma for diverse populations (including race/ethnicity, gender, age).

#### 2.2.2 Evaluation survey

To obtain the CEU certificate, individuals were required to access each section of the training content, and then they were required to complete a brief, anonymous online evaluation survey. The survey included 23 questions (Table 1): (a) how well each learning objective was met (12 items; ranging from 1 = not well at all to 5 = extremely well); (b) how well the speakers did (5 items; ranging from 1 = not well at all to 5 = extremely well); (c) detection of bias (yes/no); (d) how the participant heard of training and (e) four open‐ended items (what the participant liked best, how to improve the training, other topics of interest and additional comments).

**Table 1:** Survey evaluation results (*N* = 46)

	*M*	SD
Part 1 Objective 1 (impacts of trauma)	4.52	0.81
Part 1 Objective 2 (principles of trauma‐informed care)	4.57	0.58
Part 1 Objective 3 (trauma‐informed care strategies)	4.48	0.69
Part 2 Objective 1 (define patient‐centred outcomes research)	4.50	0.72
Part 2 Objective 2 (PCORI: types of stakeholders)	4.46	0.75
Part 2 Objective 3 (PCORI: benefits of engaging in PCOR)	4.41	0.72
Part 3 Objective 1 (engaging in trauma‐informed PCOR)	4.39	0.71
Part 3 Objective 2 (collaborating on trauma‐informed PCOR)	4.57	0.62
Speakers performed well	4.57	0.58
Speakers used effective methods	4.48	0.69
Speakers were clear and understandable	4.63	0.57
Speakers provided accurate information	4.74	0.49
Speakers provided current information	4.74	0.49
Speakers used time wisely	4.67	0.60

	*N*	%
Bias detected (no)	45	97.83
How learn about training		
Organization where I work	22	47.83
Community/advocacy organization	7	15.22
Colleague or friend	6	13.04
Professional organization of which I am a member	3	6.52
Other	8	17.39

*Note*: Means are based on a 5‐point scale, ranging from 1 = not well at all to 5 = extremely well.

Abbreviations: PCOR, patient‐centred outcomes research; PCORI, Patient‐Centered Outcomes Research Institute.

#### 2.2.3 Dissemination

Brief emails and a flyer were disseminated by all partners to their contacts in Florida, such as by email, listservs and social media. Partners had contacts with a variety of networks, including the academic units of all faculty partners, regional crisis centre, statewide network of primary care providers, regional network of over 100 behavioural health provider agencies, National Alliance on Mental Illness (NAMI) Florida and other mental health advocacy contacts. A news story was aired in which the first author was interviewed. These dissemination efforts took place periodically from spring to fall of 2021, resulting in 46 individuals who completed the online training and evaluation survey between March and December 2021. The university's IRB provided approval for the dissemination of the anonymous evaluation results reported herein.

#### 2.2.4 Data analyses

Descriptive statistics were calculated for all closed‐ended survey items. For the open‐ended questions, the first two authors used an inductive method to determine themes.^54^ Independently, the two authors analysed and categorized responses from the open‐ended responses. Next, the two authors discussed, reviewed and refined the categories into final themes, discussing until they resolved discrepancies. Regarding reflection activities, the quantitative and qualitative results were presented at a monthly partnership meeting, with designated time for discussion, after which a draft of this manuscript was distributed to all partners with 1 week to review and submit written feedback or discuss with the first author. Following the submission of the manuscript to this journal, the process was repeated: journal reviewers' feedback was reviewed and discussed at a monthly partnership meeting, the first author revised the manuscript and cover letter and all partners had 1 week to review and submit feedback.

## 3 RESULTS

Forty‐six individuals completed the online training and evaluation survey. As shown in Table 1, participants' ratings for the achievement of the learning objectives and speakers' performance were very positive, with average ratings ranging from 4.39 to 4.74 on the 5‐point scale. One individual detected bias; 45 did not. Most participants learned about the training from their work setting (*n* = 22, 47.8%).

The main themes of the open‐ended comments (Table 2) were largely positive, with the most salient theme being that it was informative (*n* = 12; e.g., ‘It was quite informative and delivered in an easy to absorb manner. Plus it applies real‐world examples to the concepts’). Another salient theme involved the impact of the lived experiences shared by patient partners (*n* = 10; e.g., ‘What I liked best was the patient feedback and sharing of experiences’.). Most participants commented that they did not have suggestions for improvements (*n* = 15), with the most common suggestions involving technical improvements (*n* = 8; e.g., ‘The sound was not consistent and I had to keep making it louder or softer depending on the video’.), desiring more detailed information (*n* = 6; e.g., ‘Suggestion of more tools that can be used with patient care’) and suggesting more interactive exercises (*n* = 5; e.g., ‘more activities to complete after each section’). Similarly, most participants commented that they did not have other suggestions for topics (*n* = 17); the most common suggestion related to wanting more details regarding trauma interventions (*n* = 10; e.g., ‘I would like more practice‐based examples of how to work with patients who have experienced trauma’). Last, most participants commented that they did not have additional input (*n* = 22), with the most common final comments involving general positive feedback (*n* = 16; e.g., ‘Thank you for providing me with such great knowledge!’).

**Table 2:** Main themes of open‐ended responses (*N* = 46)

	*n*
What liked best	
Informative	12
Lived experiences	10
Trauma information	5
Easy to access	4
Visuals	4
Self‐paced format	4
Trauma‐informed care research	2
Applicability of information	1
Patient‐centred focus	1
Different perspectives	1
Did not respond	2
How improve	
No suggestions	15
Technical improvements	8
More detailed information	6
Interactive content	5
Navigating Part 2 readings	3
Shorten	3
Other	3
Did not respond	3
Other topics	
No suggestions	17
Trauma interventions	10
Other relevant health topics	3
Involving patients in research	2
Mental health	2
Patient advocacy	2
Self‐care	2
Other	3
Did not respond	5
Additional comments	
None	22
General positive comments	16
Learning how to get involved	2
Other	2
Did not respond	4

## 4 DISCUSSION

This article describes the development and evaluation of training about TIC and its application to the co‐production of research by the patient and professional stakeholders. The experience of the *my*PATH partnership indicates that it is possible to integrate TIC principles into a research partnership's processes, and feedback from 46 training participants outside the partnership was largely positive. Training participants perceived that the learning objectives were met; they particularly valued the informative nature of the training and personal statements based on individuals' lived experiences. The inclusion of partners' lived experiences related to traumatic experiences and co‐producing research seemed to help trainees relate better to the academic material or to see how the ideas could be realistically applied. The most salient suggestions for improvements related to technical matters and the desire for additional details, particularly related to trauma interventions. The primary limitations of the current evaluation include the relatively small number of participants, the lack of information regarding participants' backgrounds and the lack of data regarding real‐world impacts, such as how the training may have impacted participants' work or collaborations. These limitations could be addressed in future research to evaluate the training with other research partnerships. It would be valuable to incorporate short‐term and longer‐term follow‐up and to evaluate real‐world impacts; hypothesized impacts of this training include improving the engagement of partners with lived experience of trauma in PPIE‐based research partnerships, improving the functioning within such partnerships, increasing the number of partnerships that incorporate trauma into their research and improving how stakeholders interact with trauma survivors in their regular work.

In addition to future research on training, the *my*PATH partnership continues to apply these TIC‐based research strategies to ongoing initiatives. Partners have reviewed and updated the governance document as new partners have been added, to emphasize the importance of thorough review before committing to joining the partnership. Regarding research endeavours, the partnership has conducted online surveys and qualitative interviews with professionals and patients regarding COVID‐19, trauma and telehealth, and has submitted a research proposal involving TIC and other interventions for primary care patients with trauma. This proposal was informed by input from training participants and other professional and patient representatives in Florida through an online survey (*N* = 249). The proposed study design emphasized inclusion, such as applying broad inclusion criteria (posttraumatic stress symptoms, not only PTSD), including English‐ and Spanish‐speaking patients, and including primary care clinics across different geographical regions that serve diverse patients (e.g., a range of racial and ethnic groups, urban/rural, LGBTQ+, older adults). We also have developed a listserv and quarterly newsletter including brief information about topics requested from the training feedback, such as trauma, trauma interventions, TIC and relevant research. The listserv and newsletter are also avenues to solicit feedback on future research products and plans.

## 5 CONCLUSION

To summarize, traumatic experiences and their impacts are very common among individuals with a wide range of physical and behavioural health conditions, and trauma is likely relevant to many partnerships that are co‐producing health‐related research, both in terms of the health condition being studied as well as personal relevance for many stakeholders who engage with the group. Our partnership developed training that reviews trauma, TIC and stakeholder‐driven research, and that applies TIC principles to stakeholder‐driven research. Training participants perceived that the learning objectives were met, and they seemed to appreciate the combination of academic and personal perspectives and desire additional training on these topics.

It is recommended that research partnerships consider learning more about trauma, how trauma relates to the health condition under study, TIC and how TIC principles could be applied to their partnership's work. Many research partnerships may already be implementing strategies that foster some TIC principles, given their overlap with general principles of research co‐production. For example, PCORI has identified four categories of engagement principles: reciprocal relationships (defining roles and decision‐making collaboratively); co‐learning (about the research process, content and research engagement); partnerships (fairness in compensation and expectations, commitment to diversity and cultural competence) and transparency, honesty and trust.^53^ Several of these principles overlap with TIC, such as fostering collaboration and empowerment of all stakeholders, building transparency and trustworthiness and attending to cultural diversity and competence. TIC principles also include explicitly attending to safety, such as discussing how trauma will be conceptualized and discussed among the partners and paying special attention to the empowerment of all partners, including recognizing the blurred boundaries between ‘patient’ and ‘professional’ partners regarding traumatic experiences and impacts.

There are numerous resources for learning more about trauma and TIC, including seminal SAMHSA publications^24^ and the Center for Health Care Strategies' TIC Implementation Resource Center.^46^ The TIC Implementation Resource Center is focused on implementing TIC in healthcare settings and has a wide range of resources, ranging from introductory material to detailed implementation guidance. To learn more about strategies for applying TIC principles to research co‐production, the *my*PATH strategies (Figure 1) and training is the only resource of which we are aware. One article was recently published (after our training) that described the application of TIC to a research advisory board of women who had experienced intimate partner violence,^42^ describing similar strategies as *my*PATH.

For interested research partnerships, it is recommended to begin discussing trauma in a general sense, and then if the partnership agrees, preview training options, select training and other resources together, participate in selected training together and then discuss how the content and strategies relate and could be incorporated into their partnership's processes and work. Potential benefits of implementing the *my*PATH strategies or learning more about trauma and TIC, in general, include a greater sense of safety, empowerment, mutual understanding and engagement among stakeholders involved in co‐producing research, as well as producing research that is more relevant to patients and providers in the real world, by addressing the impacts of trauma for the health condition under study.

## AUTHOR CONTRIBUTIONS

Amber M. Gum, PhD, directs the *my*PATH Partnership and led the development of the partnership, training, training evaluation and preparation of this manuscript. Mary Goldsworthy, MPH, is a *my*PATH Partner and was a Project Coordinator who facilitated partnership development, contributed significantly to the preparation of the training content and presentations, conducted qualitative analyses for this manuscript and reviewed a draft manuscript. Lucy Guerra, MD, MPH, is a *my*PATH Partner who contributed significantly to the development of the partnership, contributed significantly to the preparation of the training content and presentations and reviewed a draft manuscript. Alison Salloum, PhD, is a *my*PATH Partner who contributed significantly to the development of the partnership, contributed significantly to the preparation of the training content and presentations and reviewed a draft manuscript. Meredith Grau, MS, is a *my*PATH Partner who contributed significantly to the development of the partnership, contributed significantly to the preparation of the training content and presentations and reviewed a draft manuscript. Sheri Gottstein, BA, RN, is a *my*PATH Partner who contributed significantly to the development of the partnership, contributed significantly to the preparation of the training content and presentations and reviewed a draft manuscript. Carol Horvath, is a *my*PATH Partner who contributed significantly to the development of the partnership, contributed significantly to the preparation of the training content and presentations and reviewed a draft manuscript. Annanora Fields, BA, is a *my*PATH Partner who contributed significantly to the development of the partnership and reviewed a draft manuscript. Johnny Crowder, BA, is a *my*PATH Partner who contributed significantly to the development of the partnership, contributed significantly to the preparation of the training content and presentations and reviewed a draft manuscript. Robb Holley, MD, is a *my*PATH Partner who worked as a Project Coordinator and facilitated partnership development and infrastructure and reviewed a draft manuscript. Leigh J. Ruth, MD, is a *my*PATH Partner who contributed significantly to the development of the partnership and reviewed a draft manuscript. Karim Hanna, MD, is a *my*PATH Partner who contributed significantly to the development of the partnership and reviewed a draft manuscript.

## CONFLICT OF INTEREST

The authors declare no conflict of interest.

## Data Availability

De‐identified data from the evaluation results are available from the first author upon request.
